# Beyond antimicrobials: a host-directed precision pharmacotherapy framework for septic shock

**DOI:** 10.3389/fmed.2026.1811347

**Published:** 2026-04-23

**Authors:** Xian-zhi Cheng, Jia-shu Li, Ping An, Bing Han, Li-kun Li, Kong-lei Ma, Hong Zhang

**Affiliations:** 1 Department of Pharmacy, Hongqi Hospital Affiliated to Mudanjiang Medical University, Mudanjiang, China; 2 Department of Critical Care Medicine, Hongqi Hospital Affiliated to Mudanjiang Medical University, Mudanjiang, China; 3 School of Clinical Medicine, Mudanjiang Medical University, Mudanjiang, China; 4 School of Pharmacy, Mudanjiang Medical University, Mudanjiang, China

**Keywords:** biomarkers, cost-effectiveness analysis, endotyping, host-directed therapy, precision medicine, real-time monitoring, septic shock

## Abstract

Despite progress in antibiotic therapy and supportive care, septic shock continues to pose a substantial mortality burden in intensive care units, highlighting persistent limitations in current pathogen-focused treatment approaches. A central issue is the heterogeneity of host immune responses to infection, manifesting as diverse clinical phenotypes—such as immunosuppression and hyperinflammation—that drive variability in outcomes. Thus, evolving the therapeutic paradigm from infection control alone to precise modulation of the host response represents a critical next step for improving prognosis. This perspective article scrutinizes an integrated precision medicine framework that combines multidimensional host endotyping, targeted therapeutic strategies, and real-time monitoring with dynamic feedback. We examine its scientific foundations, potential clinical applications, and implementation pathways, while addressing current challenges and future directions. The goal is to offer a structured perspective and actionable insights to advance individualized management of septic shock.

## 1 Introduction

Septic shock is a primary cause of death in intensive care units globally, presenting a significant epidemiological burden with persistently high mortality rates (1). The current standard treatment—comprising early empirical antibiotics, prompt source control, and hemodynamic resuscitation with fluids and vasopressors—has substantially improved initial patient management (2). Despite these advances, a considerable residual risk remains (3). Many patients continue to deteriorate or progress into a state of prolonged critical illness even after initial infection control, highlighting the limitations of current therapies in securing long-term recovery (4).

The central challenge lies in the marked heterogeneity of the host immune response (5). Although infection initiates the process, the subsequent dysregulated immune and inflammatory reactions critically influence organ injury and recovery. Clinically, patients often diverge into distinct immune phenotypes: some develop “immune paralysis,” characterized by lymphopenia and impaired immune function, which increases vulnerability to secondary infections; others experience persistent, dysregulated inflammation, often termed “persistent inflammation, immunosuppression, and catabolism syndrome,” leading to ongoing tissue injury (6, 7). This imbalance in host homeostasis represents a key independent determinant of treatment response variability and clinical outcomes (5, 6).

Previous attempts to modulate the host response using a uniform approach—such as administering single immunomodulatory agents targeting either excessive inflammation or immunosuppression—have largely failed in clinical trials (8). These outcomes underscore that simplistic, one-directional interventions are inadequate and potentially harmful (9). Effective therapy should instead account for the dynamic and individualized nature of immune dysfunction. Therefore, moving from a generalized to a precision-based strategy is essential for the success of future host-directed treatments (10, 11).

Despite growing interest in immunomodulatory therapies for sepsis, the clinical translation of many targeted interventions—including granulocyte-macrophage colony-stimulating factor, interferon-*γ*, and immune checkpoint inhibitors—has yielded inconsistent or inconclusive results (12). Rather than reflecting an inherent lack of therapeutic efficacy, these outcomes expose a critical disconnect between mechanistic rationale and clinical implementation (6, 13). Specifically, current intervention strategies often fail to align with the dynamic and heterogeneous nature of host responses, including temporal shifts in immune status and variability across patient subgroups (14). This misalignment suggests that the limitations of prior trials are rooted not only in therapeutic design, but also in the absence of biologically informed stratification and adaptive treatment frameworks (9).

**Figure 1:**
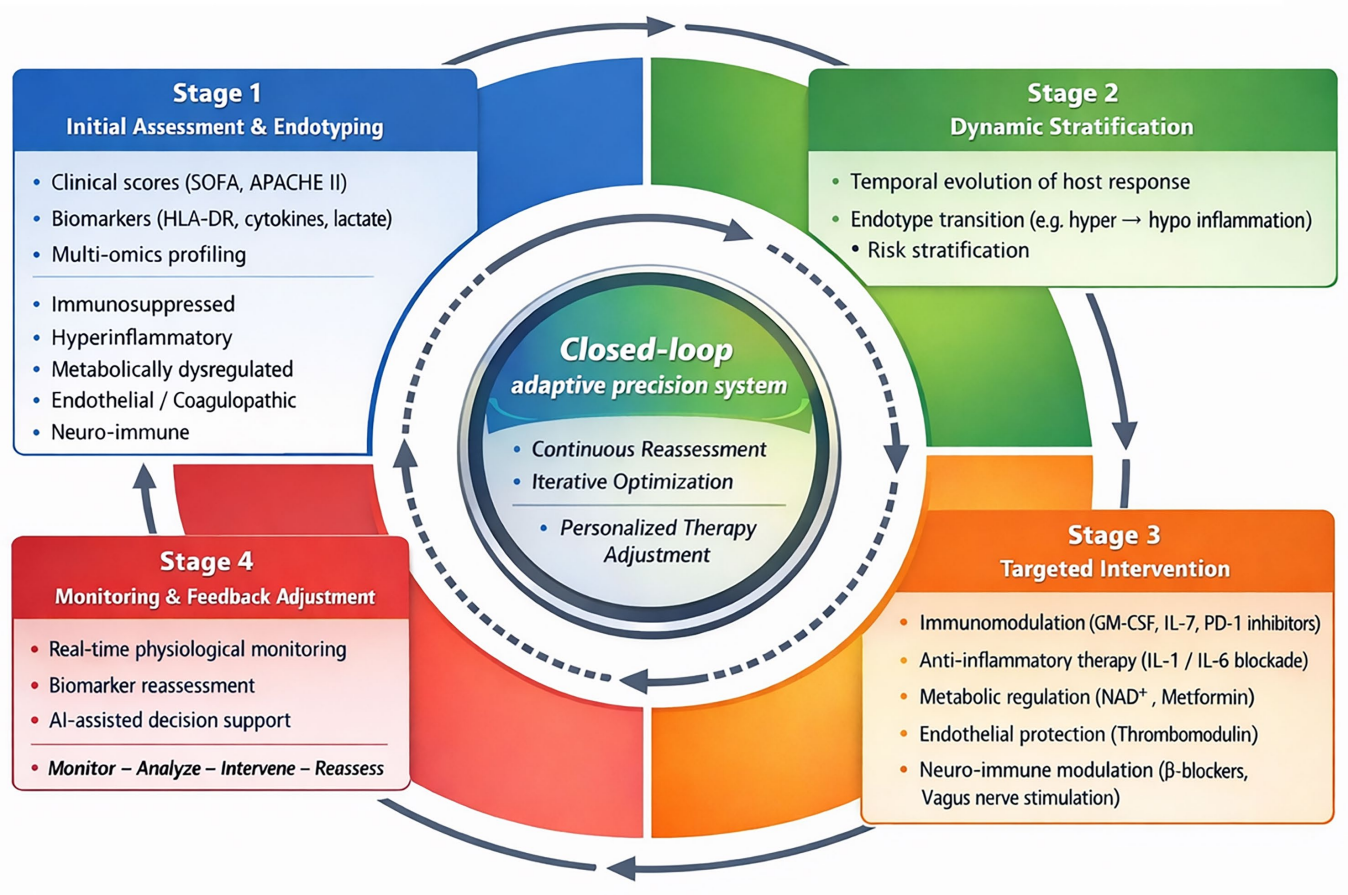
Integrated host-directed precision pharmacotherapy framework for septic shock: a dynamic closed-loop system.

Taken together, these limitations highlight the need for a paradigm shift in sepsis management. This perspective article proposes an integrated precision medicine framework to advance host-directed therapy in septic shock. First, we scrutinize available biomarkers and immune monitoring tools for defining host response endotypes. Next, we discuss how dynamic immune phenotyping can guide patient stratification. Finally, we examine the potential for aligning targeted therapies with specific immune subtypes and outline ongoing challenges. Through this framework, we aim to support the transition from a pathogen-centered to a patient-tailored therapeutic paradigm in sepsis management (Figure 1).

## 2 Multi-dimensional host homeostasis endotyping and biomarkers

### 2.1 Beyond clinical manifestations: biology-based homeostasis endotyping

Clinical management of sepsis has traditionally relied on physiological parameters and organ function scores. However, patients with similar clinical presentations often show markedly different treatment responses and outcomes, a disparity rooted in their underlying biological heterogeneity (15). Importantly, it is necessary to distinguish between the concepts of “subphenotype” and “endotype,” which are often used interchangeably but represent fundamentally different levels of disease classification (Box 1) (16, 17). While subphenotypes are typically defined based on observable clinical characteristics or trajectories, endotypes are grounded in distinct biological mechanisms and pathophysiological processes (16–18). This conceptual distinction is critical for advancing precision medicine, as it shifts the focus from descriptive classification toward mechanism-based stratification.

BOX 1 **Conceptual distinction between “endotype” and “subphenotype”**

In precision medicine, subphenotypes refer to clinically observable groupings based on shared clinical characteristics, trajectories, or outcomes. In contrast, endotypes are defined by distinct underlying biological mechanisms, typically identified through multi-omics profiling and pathophysiological pathways.

In this framework, “host homeostasis endotyping” specifically refers to biologically driven stratification based on mechanistic dysregulation (e.g., immune, metabolic, or endothelial pathways), rather than clinical manifestations alone.

Advances in multi-omics technologies and single-cell analysis now enable the classification of host homeostasis based on distinct biological mechanisms (17). Integration of genomics, transcriptomics, proteomics, and metabolomics data has identified specific sepsis endotypes, including immunosuppressive, hyperinflammatory, metabolically dysregulated, and coagulopathic subtypes (19). These endotypes extend beyond symptomatic description to reveal the precise pathophysiological pathways driving disease progression. Critically, host homeostasis is not static but evolves dynamically during illness and in response to therapy—for example, shifting from early hyperinflammation to late immunosuppression (20). Effective endotyping should therefore capture and reflect this temporal progression.

### 2.2 Toolkit for precision endotyping

Implementing precise endotyping requires an integrated toolkit spanning biomarker discovery to clinical application (21). Promising biomarkers reflect diverse aspects of host response: monocyte HLA-DR expression indicates immunosuppression; cytokine profiles differentiate inflammatory states; PD-1/PD-L1 levels signal T-cell exhaustion; and metabolites such as lactate reflect metabolic stress (22–24). However, most biomarkers remain in research phases, limited by challenges in standardization and clinical validation. Future progress depends on developing rapid, reliable point-of-care assays to translate endotyping into real-time clinical guidance (25).

To further enhance clinical applicability, a pragmatic approach to endotyping should incorporate a structured, minimal biomarker panel that captures key biological domains while remaining feasible for bedside implementation (26, 27). Such a panel may include representative markers of immune function (e.g., monocyte HLA-DR, interleukin-6, PD-1/PD-L1), metabolic status (e.g., lactate, glucose variability), and endothelial or coagulation dysfunction (e.g., angiopoietin-2, D-dimer) (19, 28). Rather than relying on exhaustive multi-omics profiling, this “minimum viable panel” strategy prioritizes translational feasibility and scalability across diverse clinical settings (19).

Although universally accepted clinical thresholds have not yet been established, emerging evidence supports the use of semi-quantitative interpretation frameworks. For instance, markedly reduced HLA-DR expression may indicate a state of immunosuppression, whereas persistently elevated pro-inflammatory cytokines such as interleukin-6 may suggest a hyperinflammatory phenotype (22, 29). These approximated biological boundaries, while not definitive, can provide an initial decision-support scaffold for early-stage clinical application and biomarker-guided trial design, pending rigorous prospective validation.

To maximize clinical utility, biological endotyping should be combined with established clinical assessment tools. While scores such as SOFA and APACHE II quantify illness severity and prognosis, they do not elucidate underlying mechanisms (16). Integrating these clinical scores with molecular biomarkers or endotypic profiles can create more powerful, multi-dimensional stratification models (15). Such an approach would improve risk prediction and, importantly, help identify patients most likely to respond to targeted therapies such as immunomodulators (30). This synthesis of clinical and biological data is essential for advancing personalized, host-directed treatment strategies in sepsis (31).

## 3 Precision therapeutic arsenal targeting host pathways

The ultimate aim of precisely identifying host homeostasis endotypes is to guide highly selective, targeted treatments. In contrast to earlier “one-size-fits-all” immunomodulatory interventions, contemporary host-directed therapy seeks to align specific treatments with a patient’s distinct biological profile, forming a precision-guided therapeutic arsenal. To enhance transparency and reduce the risk of overestimating therapeutic effects, all interventions discussed in this section are categorized based on the strength of supporting evidence, using principles adapted from the Grading of Recommendations Assessment, Development and Evaluation framework. Evidence is stratified into four levels: high-quality randomized controlled trials, early-phase or exploratory clinical studies, secondary analyses of existing trials, and preclinical investigations. This classification is summarized in Table 1.

**Table 1:** Alignment of host endotypes with representative biomarkers, targeted therapeutic strategies, monitoring priorities, and level of evidence.

Endotype	Representative biomarkers	Therapeutic strategy	Monitoring focus	Evidence level
Immunosuppressed	↓HLA-DR, ↑PD-1	GM-CSF; IFN-γ	Immune recovery; monocyte function	RCT/clinical studies
		IL-7	Lymphocyte restoration	Phase II RCT
		Anti-PD-1/PD-L1	T-cell function	Early-phase clinical trials
Hyperinflammatory	IL-6; ferritin	IL-1 blockade	Cytokine suppression	Secondary analysis of RCT
		IL-6 blockade	Inflammation control	Clinical studies
Metabolically dysregulated	Lactate; insulin resistance	NAD^+^ precursors	Mitochondrial function	Preclinical
		Metformin	Glucose metabolism	Observational/meta-analysis
		Rapamycin analogs	Autophagy regulation	Preclinical
Endothelial/coagulopathic	Ang-2; D-dimer	Recombinant thrombomodulin	Coagulation status	Phase II RCT
		Endothelial stabilizers	Vascular integrity	Preclinical/early clinical
Neuro-immune	HRV; catecholamines	β-blockers	Cardiac efficiency	RCT
		Cholinergic agonists	Anti-inflammatory reflex	Preclinical
		Vagus nerve stimulation	Neuro-immune modulation	Early translational

HLA-DR, human leukocyte antigen-DR isotype; PD-1, programmed cell death protein 1; GM-CSF, granulocyte-macrophage colony-stimulating factor; IFN-γ, interferon-gamma; IL-7, interleukin-7; IL-1, interleukin-1; IL-6, interleukin-6; NAD^+^, nicotinamide adenine dinucleotide; Ang-2, angiopoietin-2; HRV, heart rate variability.

In this table, directional arrows indicate relative changes in biomarker levels or expression: “↑” denotes upregulation or increased expression, whereas “↓” denotes downregulation or decreased expression. Specifically, ↑PD-1 refers to increased expression of programmed cell death protein 1, which is associated with T-cell exhaustion and functional immunosuppression, rather than enhanced immune activation.

Evidence levels are categorized as follows: RCT: randomized controlled trials; Phase II RCT: early-phase randomized trials; Clinical studies: non-randomized or exploratory clinical studies; Secondary analysis: *post hoc* analysis of existing trials; Observational/meta-analysis: cohort-based or pooled analyses; Preclinical: animal or in vitro studies; and Early translational: proof-of-concept or pilot human studies.

Importantly, this precision-oriented therapeutic paradigm is informed by critical insights derived from prior unsuccessful immunomodulatory trials in sepsis (14). Accumulating evidence suggests that many of these failures were not solely attributable to insufficient biological efficacy, but rather to suboptimal clinical implementation (32). Specifically, three major limitations have been identified. First, therapeutic interventions were frequently administered without alignment to the patient’s evolving immune trajectory, neglecting the dynamic transition between hyperinflammatory and immunosuppressive states (14, 33). Second, patient stratification was largely based on clinical syndromes rather than biologically defined endotypes, resulting in heterogeneous trial populations and attenuation of detectable treatment effects (16, 17). Third, most treatment strategies were inherently static, failing to adapt to the temporal evolution of host responses (9). Collectively, these limitations underscore the necessity of transitioning toward a dynamic, biology-driven, and adaptive therapeutic framework. The present model addresses these challenges by integrating real-time endotyping, biomarker-guided stratification, and iterative treatment adjustment, thereby enabling a more responsive and individualized approach to host-directed therapy.

### 3.1 Immunomodulatory strategies

Based on precise stratification of a patient’s immune state, immunomodulatory strategies can be individually tailored (31). For patients clearly classified as “immunosuppressed,” the therapeutic focus is on restoring immune-cell function and numbers. Granulocyte-macrophage colony-stimulating factor and interferon-γ may be used to enhance monocyte/macrophage phagocytic and bactericidal activity (31, 34). Interleukin-7, a key lymphocyte growth factor, holds promise for reversing T-cell exhaustion and expanding the lymphocyte pool (35). Targeting T-cell dysfunction specifically, immune checkpoint inhibitors such as anti-PD-1/PD-L1 antibodies have shown potential in selected subgroups to reverse immune paralysis (36). Conversely, for patients accurately identified as “hyperinflammatory” through biomarkers, careful use of cytokine-targeted antagonists—for instance, interleukin-1 or interleukin-6 inhibitors—may be considered to mitigate excessive inflammatory cascades and limit tissue damage, provided infection source control is assured (37) (see Table 1 for corresponding endotype-specific therapeutic strategies and evidence stratification).

### 3.2 Metabolic and cellular energy therapies

Sepsis often involves profound metabolic reprogramming and cellular energy failure. For patients with a “metabolically dysregulated” endotype, therapies aimed at improving cellular energetics and restoring homeostasis are essential (38, 39). NAD^+^ precursors such as nicotinamide riboside are designed to enhance mitochondrial function and oxidative phosphorylation, countering bioenergetic collapse (40). In patients exhibiting insulin resistance and disordered glucose metabolism, metformin, used cautiously with close monitoring, may help re-establish metabolic balance (41). Moreover, modulation of autophagy—for example via rapamycin analogs (currently supported by preclinical evidence)—could theoretically aid in clearing damaged organelles, supplying energy substrates, and regulating inflammation, although its precise timing and target population in sepsis remain to be clearly defined (42) (see Table 1 for alignment of metabolic-targeted therapies with evidence levels).

### 3.3 Endothelial and coagulation pathway modulation

Disseminated intravascular coagulation and endothelial dysfunction are central to impaired organ perfusion (43). In subgroups with marked coagulation activation and endothelial injury, anticoagulation should advance beyond conventional heparin-based approaches toward personalized strategies (44). Recombinant agents targeting thrombomodulin can potentiate the natural protein C anticoagulant and cytoprotective pathways (44). Antagonists of angiopoietin-2, a key regulator of vascular permeability, may stabilize the endothelial barrier and reduce tissue edema (45). The choice of such interventions should be guided by real-time, patient-specific molecular profiles of coagulation and endothelial injury markers (46) (see Table 1 for corresponding endothelial/coagulopathic endotype interventions and supporting evidence).

### 3.4 Neuro-immune modulation and autonomic regulation

The neuro-immune axis plays an important role in sepsis pathophysiology. Pharmacological modulation of the cholinergic anti-inflammatory pathway—for instance via selective cholinergic receptor agonists—or bioelectronic techniques offer novel means to dampen excessive inflammation (47). Additionally, sepsis-related myocardial depression is associated with excessive sympathetic activation. In specific patients with persistent tachycardia and impaired cardiac function, judicious use of β-blockers may improve cardiac efficiency, lower metabolic demand, and potentially confer beneficial immunomodulatory effects through influences on immune-cell activity (48, 49). Success of these approaches depends entirely on a deep, dynamic understanding and monitoring of the interplay among the patient’s neuro-immune-cardiovascular states (50) (see Table 1 for neuro-immune modulation strategies and evidence classification).

## 4 Real-time monitoring and feedback-adjusted therapeutic system

While host homeostasis endotyping and targeted therapies establish the basis for precision treatment, the patient’s pathophysiological state is dynamic, evolving continuously throughout the disease course and in response to therapeutic interventions (51). Thus, a robust precision medicine framework should incorporate a third, equally critical pillar: an intelligent therapeutic system designed for real-time monitoring and dynamic, feedback-guided adjustment. This system aims to transition from the traditional, static “diagnose-and-treat” paradigm to a closed-loop management process characterized by continuous sensing, intelligent analysis, and adaptive optimization (52).

### 4.1 Dynamic monitoring technologies

Enabling real-time feedback first requires access to continuous, non-invasive or minimally invasive dynamic monitoring tools. At the physiological level, artificial intelligence-based analysis of continuous cardiovascular waveforms goes beyond conventional blood-pressure monitoring, detecting subtle, real-time changes in cardiac output, vascular tone, and fluid responsiveness to guide resuscitation strategies (53). Point-of-care ultrasound and advanced, minimally invasive hemodynamic monitoring permit dynamic assessment of organ perfusion and cardiac function, providing immediate evaluation of therapeutic responses to fluids or vasoactive agents (54). At the molecular level, establishing a comprehensive protocol for periodic biomarker measurement is essential. Serial assessment of monocyte HLA-DR expression, for example, can quantify the depth and recovery trajectory of immunosuppression, while tracking dynamic cytokine or metabolite profiles can illuminate real-time shifts in inflammatory or metabolic homeostasis (22, 55, 56). These measurements furnish an objective basis for adjusting therapy.

### 4.2 Feedback loop and adaptive treatment algorithms (conceptual framework)

It is important to emphasize that the proposed closed-loop system should currently be regarded as a conceptual framework, as most supporting evidence derives from preclinical studies and proof-of-concept investigations, with limited validation in human clinical settings. The integration of monitoring data into actionable clinical decisions calls for the construction of an intelligent “monitor-analyze-intervene-reassess” feedback loop. Central to this system is an adaptive treatment algorithm that synthesizes real-time multimodal data (physiological, imaging, and molecular biomarkers), the patient’s baseline and evolving endotype, and individual pharmacogenomic features (e.g., drug-metabolism enzyme or receptor genotypes) (57).

From a translational perspective, this closed-loop architecture is not merely a technical construct but a response to well-recognized limitations in prior immunomodulatory trials (29). By enabling continuous reassessment of patient-specific endotypes and facilitating real-time modification of therapeutic strategies, the framework reduces reliance on static, one-time treatment decisions that have historically contributed to inconsistent clinical outcomes (16, 28, 33). In this sense, the integration of dynamic monitoring with adaptive algorithms represents a conceptual and operational shift toward temporally responsive and biologically aligned precision therapy.

To enhance translational feasibility while acknowledging the complexity of full-scale multi-omics integration, an initial implementation strategy may adopt a “minimum viable dataset” approach (58). This pragmatic model prioritizes the integration of three core data domains: (1) essential laboratory biomarkers reflecting immune, metabolic, and endothelial states; (2) continuous physiological monitoring data capturing dynamic organ function; and (3) standardized clinical severity scores, such as the SOFA score. By focusing on high-yield, readily accessible variables, this approach reduces implementation barriers and enables early adoption in resource-constrained settings (51, 59), while preserving compatibility with future expansion toward more comprehensive, AI-driven multimodal systems.

To move beyond a purely conceptual model, a pragmatic clinical decision-support workflow can be embedded within this framework. Specifically, an initial rapid biomarker assessment enables classification of patients into dominant biological endotypes, such as immunosuppressed, hyperinflammatory, metabolically dysregulated, or endothelial dysfunction phenotypes (16, 60). Based on this classification, targeted therapeutic strategies can be aligned with the identified endotype—for example, immunostimulatory approaches for immunosuppressed patients or anti-inflammatory interventions for those exhibiting hyperinflammatory profiles (61). Treatment should then be initiated within predefined safety and monitoring boundaries. Crucially, this process requires iterative reassessment of key biomarkers at regular intervals (e.g., every 24–48 h), allowing clinicians to capture the dynamic trajectory of the host response (62). Therapeutic strategies can subsequently be adjusted, escalated, or de-escalated according to these evolving patterns. This iterative “classify–treat–reassess” process operationalizes the closed-loop concept and provides a feasible entry point for early-stage clinical implementation of precision medicine in septic shock.

Within this framework, machine learning and artificial intelligence play a pivotal role. These technologies can integrate large-scale, heterogeneous time-series data, recognize complex nonlinear patterns, and execute three core functions: first, predicting a patient’s near-term response and potential risks under the current treatment plan to enable early warning (63); second, recommending optimized, personalized interventions—such as drug selection, dosing, or timing—based on the continuously updated clinical picture (64); and third, iteratively refining the algorithm through continuous learning, allowing the model to evolve as clinical data accumulate (65). Collectively, these capabilities support the development of an intelligent decision-support system capable of dynamic regimen optimization and personalized dose titration.

However, several critical challenges should be addressed before such systems can be translated into routine clinical practice. First, the integration of multimodal data streams requires robust digital infrastructure, standardized data acquisition protocols, and interoperable data platforms to ensure reliability and scalability (66). Second, issues related to algorithmic bias, model transparency, and external validity should be rigorously evaluated to ensure safe and equitable application across diverse patient populations (67, 68). Third, clinician acceptance, interpretability, and usability of bedside decision-support systems represent key determinants of successful implementation, underscoring the importance of user-centered design (69). Accordingly, future research should prioritize prospective clinical validation, real-world implementation studies, and the development of human-centered interfaces to bridge the gap between computational innovation and bedside applicability.

## 5 Clinical implementation pathways and challenges

To translate this precision medicine framework for sepsis into clinical practice, a defined roadmap is essential to bridge current knowledge and bedside application, while comprehensively addressing the multifaceted challenges of implementation.

### 5.1 Translational roadmap from research to clinic

Implementation should follow a phased and stepwise strategy (70, 71). First, the proposed host endotyping system should be rigorously validated in large, high-quality, multicenter retrospective cohorts to confirm its association with clinical outcomes and treatment response (72). Second, prospective biomarker-guided Phase II trials should be initiated, wherein patients are stratified by predefined biomarkers to assess the preliminary safety and biological activity of endotype-targeted therapies (73, 74). The third and most definitive phase involves conducting adaptive randomized controlled trials based on host endotypes (70, 71). Trial designs may include “basket” trials, evaluating multiple distinct therapies within a single endotype (e.g., immunosuppressed), or “umbrella” trials, which assign patients with different endotypes (e.g., hyperinflammatory or metabolically dysregulated) to matched treatment arms within a unified platform. Such adaptive designs offer an efficient method to validate the efficacy of precision endotype-guided treatment.

### 5.2 Major challenges and countermeasures

Several key challenges should be addressed. Technically, barriers include the rapid generation, standardized interpretation, and high cost of multi-omics data (75). Countermeasures involve developing simplified, cost-effective point-of-care or near-patient testing panels, alongside shared databases with standardized analytical protocols. Scientifically, the spatiotemporal heterogeneity of host responses—variation across time and tissues—and the redundancy of pathological pathways require dynamic and potentially combinatorial therapeutic strategies (76). Clinically and ethically, the complexity of endotype-based adaptive trials places high operational demands on clinical sites. Personalized treatment approaches should also be integrated with evidence-based standard guidelines (77). Furthermore, proactive consideration of resource accessibility and health equity is critical to ensure that precision medicine advances benefit broad populations and do not widen existing healthcare disparities (78, 79).

### 5.3 Cost-effectiveness considerations and implementation strategy

While formal health economic evaluations are not yet available, the proposed precision framework holds promise for improving cost-effectiveness (80). By enabling targeted interventions and reducing the use of ineffective or potentially harmful treatments, endotype-guided strategies may enhance the efficiency of resource allocation (81).

Nevertheless, implementation entails considerable upfront costs, including those associated with multi-omics profiling, biomarker assays, and digital infrastructure (82). A phased implementation approach may therefore be warranted, with initial focus on high-risk populations most likely to benefit (82, 83). To support future adoption, rigorous health economic assessments—including incremental cost-effectiveness ratio analyses and budget impact evaluations—are needed to determine the economic viability of this precision-based paradigm.

## Data Availability

The original contributions presented in the study are included in the article/supplementary material, further inquiries can be directed to the corresponding author.

## References

[1] Fleischmann-Struzek C Mellhammar L Rose N Cassini A Rudd KE Schlattmann P . Incidence and mortality of hospital- and ICU-treated sepsis: results from an updated and expanded systematic review and meta-analysis. Intensive Care Med. (2020) 46:1552–62. doi: 10.1007/s00134-020-06151-x, 32572531 PMC7381468

[2] Evans L Rhodes A Alhazzani W Antonelli M Coopersmith CM French C . Surviving sepsis campaign: international guidelines for management of sepsis and septic shock 2021. Intensive Care Med. (2021) 47:1181–247. doi: 10.1007/s00134-021-06506-y 34599691 PMC8486643

[3] Prescott HC Angus DC. Enhancing recovery from Sepsis: a review. JAMA. (2018) 319:62–75. doi: 10.1001/jama.2017.17687, 29297082 PMC5839473

[4] Hawkins RB Raymond SL Stortz JA Horiguchi H Brakenridge SC Gardner A . Chronic critical illness and the persistent inflammation, immunosuppression, and catabolism syndrome. Front Immunol. (2018) 9:1511. doi: 10.3389/fimmu.2018.01511, 30013565 PMC6036179

[5] Wiersinga WJ van der Poll T. Immunopathophysiology of human sepsis. EBioMedicine. (2022) 86:104363. doi: 10.1016/j.ebiom.2022.104363, 36470832 PMC9783164

[6] Hotchkiss RS Monneret G Payen D. Sepsis-induced immunosuppression: from cellular dysfunctions to immunotherapy. Nat Rev Immunol. (2013) 13:862–74. doi: 10.1038/nri3552, 24232462 PMC4077177

[7] Mira JC Brakenridge SC Moldawer LL Moore FA. Persistent inflammation, immunosuppression and catabolism syndrome. Crit Care Clin. (2017) 33:245–58. doi: 10.1016/j.ccc.2016.12.001, 28284293 PMC5351769

[8] Abraham E Wunderink R Silverman H Perl TM Nasraway S Levy H . Efficacy and safety of monoclonal antibody to human tumor necrosis factor alpha in patients with sepsis syndrome. A randomized, controlled, double-blind, multicenter clinical trial. JAMA. (1995) 273:934–41. doi: 10.1001/jama.1995.03520360048038 7884952

[9] Marshall JC. Why have clinical trials in sepsis failed? Trends Mol Med. (2014) 20:195–203. doi: 10.1016/j.molmed.2014.01.007 24581450

[10] Leventogiannis K Kyriazopoulou E Antonakos N Kotsaki A Tsangaris I Markopoulou D . Toward personalized immunotherapy in sepsis: the PROVIDE randomized clinical trial. Cell Rep Med. (2022) 3:100817. doi: 10.1016/j.xcrm.2022.100817, 36384100 PMC9729870

[11] van Peters Ton AM Kox M Abdo WF Pickkers P. Precision immunotherapy for sepsis. Front Immunol. (2018) 9:1926. doi: 10.3389/fimmu.2018.01926, 30233566 PMC6133985

[12] Hotchkiss RS Opal S. Immunotherapy for sepsis--a new approach against an ancient foe. N Engl J Med. (2010) 363:87–9. doi: 10.1056/NEJMcibr1004371, 20592301 PMC4136660

[13] Shankar-Hari M Phillips GS Levy ML Seymour CW Liu VX Deutschman CS . Developing a new definition and assessing new clinical criteria for septic shock: for the third international consensus definitions for Sepsis and septic shock (Sepsis-3). JAMA. (2016) 315:775–87. doi: 10.1001/jama.2016.0289, 26903336 PMC4910392

[14] van der Poll T Shankar-Hari M Wiersinga WJ. The immunology of sepsis. Immunity. (2021) 54:2450–64. doi: 10.1016/j.immuni.2021.10.012, 34758337

[15] Davenport EE Burnham KL Radhakrishnan J Humburg P Hutton P Mills TC . Genomic landscape of the individual host response and outcomes in sepsis: a prospective cohort study. Lancet Respir Med. (2016) 4:259–71. doi: 10.1016/S2213-2600(16)00046-1, 26917434 PMC4820667

[16] Seymour CW Kennedy JN Wang S Chang CH Elliott CF Xu Z . Derivation, validation, and potential treatment implications of novel clinical phenotypes for sepsis. JAMA. (2019) 321:2003–17. doi: 10.1001/jama.2019.5791, 31104070 PMC6537818

[17] Scicluna BP van Vught LA Zwinderman AH Wiewel MA Davenport EE Burnham KL . Classification of patients with sepsis according to blood genomic endotype: a prospective cohort study. Lancet Respir Med. (2017) 5:816–26. doi: 10.1016/S2213-2600(17)30294-1 28864056

[18] Prescott HC Calfee CS Thompson BT Angus DC Liu VX. Toward smarter lumping and smarter splitting: rethinking strategies for Sepsis and acute respiratory distress syndrome clinical trial design. Am J Respir Crit Care Med. (2016) 194:147–55. doi: 10.1164/rccm.201512-2544CP, 27244481 PMC5003218

[19] Balch JA Chen UI Liesenfeld O Starostik P Loftus TJ Efron PA . Defining critical illness using immunological endotypes in patients with and without sepsis: a cohort study. Crit Care. (2023) 27:292. doi: 10.1186/s13054-023-04571-x, 37474944 PMC10360294

[20] van Amstel RBE Bartek B Vlaar APJ Gay E van Vught LA Cremer OL . Temporal transitions of the hyperinflammatory and hypoinflammatory phenotypes in critical illness. Am J Respir Crit Care Med. (2025) 211:347–56. doi: 10.1164/rccm.202406-1241OC, 39642348 PMC11936145

[21] Tang G Luo Y Song H Liu W Huang Y Wang X . The immune landscape of sepsis and using immune clusters for identifying sepsis endotypes. Front Immunol. (2024) 15:1287415. doi: 10.3389/fimmu.2024.1287415, 38707899 PMC11066285

[22] Monneret G Lafon T Gossez M Evrard B Bodinier M Rimmelé T . Monocyte HLA-DR expression in septic shock patients: insights from a 20-year real-world cohort of 1023 cases. Intensive Care Med. (2025) 51:1820–32. doi: 10.1007/s00134-025-08110-w, 40986015 PMC12504386

[23] Gao X Cai S Li X Wu G. Sepsis-induced immunosuppression: mechanisms, biomarkers and immunotherapy. Front Immunol. (2025) 16:1577105. doi: 10.3389/fimmu.2025.1577105, 40364841 PMC12069044

[24] Deulkar P Singam A Mudiganti VNKS Jain A. Lactate monitoring in intensive care: a comprehensive review of its utility and interpretation. Cureus. (2024) 16:e66356. doi: 10.7759/cureus.66356, 39246930 PMC11379417

[25] Haem Rahimi M Conti F Llitjos JF Fleurie A Cerro V Venet F . Monocyte HLA-DR expression as an enrollment biomarker in sepsis clinical trials: evaluation of two sampling tubes and definition of respective clinical thresholds. Cytometry B Clin Cytom. (2023) 104:468–70. doi: 10.1002/cyto.b.22133, 37226415

[26] van Amstel RBE Rademaker E Kennedy JN Bos LDJ Peters-Sengers H Butler JM . Clinical subtypes in critically ill patients with sepsis: validation and parsimonious classifier model development. Crit Care. (2025) 29:58. doi: 10.1186/s13054-025-05256-3, 39905513 PMC11796029

[27] Chenoweth JG Brandsma J Striegel DA Genzor P Chiyka E Blair PW . Sepsis endotypes identified by host gene expression across global cohorts. Commun Med. (2024) 4:120. doi: 10.1038/s43856-024-00542-7, 38890515 PMC11189468

[28] Shin KS. Current and emerging biomarkers for sepsis: diagnostic and prognostic tools. Biomed Sci Lett. (2025) 31:225–35. doi: 10.15616/BSL.2025.31.3.225

[29] Gouel-Chéron A Allaouchiche B Guignant C Davin F Floccard B Monneret G. Early interleukin-6 and slope of monocyte human leukocyte antigen-DR: a powerful association to predict the development of sepsis after major trauma. PLoS One. (2012) 7:e33095. doi: 10.1371/journal.pone.0033095, 22431998 PMC3303782

[30] Song J Moon S Park DW Cho HJ Kim JY Park J . Biomarker combination and SOFA score for the prediction of mortality in sepsis and septic shock: a prospective observational study according to the Sepsis-3 definitions. Medicine. (2020) 99:e20495. doi: 10.1097/MD.0000000000020495, 32481464 PMC12245219

[31] Meisel C Schefold JC Pschowski R Baumann T Hetzger K Gregor J . Granulocyte-macrophage colony-stimulating factor to reverse sepsis-associated immunosuppression: a double-blind, randomized, placebo-controlled multicenter trial. Am J Respir Crit Care Med. (2009) 180:640–8. doi: 10.1164/rccm.200903-0363OC, 19590022

[32] Hotchkiss RS Moldawer LL Opal SM Reinhart K Turnbull IR Vincent JL. Sepsis and septic shock. Nat Rev Dis Primers. (2016) 2:16045. doi: 10.1038/nrdp.2016.45, 28117397 PMC5538252

[33] Hotchkiss RS Monneret G Payen D. Immunosuppression in sepsis: a novel understanding of the disorder and a new therapeutic approach. Lancet Infect Dis. (2013) 13:260–8. doi: 10.1016/S1473-3099(13)70001-X, 23427891 PMC3798159

[34] Payen D Faivre V Miatello J Leentjens J Brumpt C Tissières P . Multicentric experience with interferon gamma therapy in sepsis induced immunosuppression. A case series. BMC Infect Dis. (2019) 19:931. doi: 10.1186/s12879-019-4526-x, 31690258 PMC6833157

[35] Daix T Mathonnet A Brakenridge S Dequin PF Mira JP Berbille F . Intravenously administered interleukin-7 to reverse lymphopenia in patients with septic shock: a double-blind, randomized, placebo-controlled trial. Ann Intensive Care. (2023) 13:17. doi: 10.1186/s13613-023-01109-w, 36906875 PMC10008152

[36] Hotchkiss RS Colston E Yende S Crouser ED Martin GS Albertson T . Immune checkpoint inhibition in sepsis: a phase 1b randomized study to evaluate the safety, tolerability, pharmacokinetics, and pharmacodynamics of nivolumab. Intensive Care Med. (2019) 45:1360–71. doi: 10.1007/s00134-019-05704-z, 31576433 PMC9006384

[37] Shakoory B Carcillo JA Chatham WW Amdur RL Zhao H Dinarello CA . Interleukin-1 receptor blockade is associated with reduced mortality in sepsis patients with features of macrophage activation syndrome: reanalysis of a prior phase III trial. Crit Care Med. (2016) 44:275–81. doi: 10.1097/CCM.0000000000001402, 26584195 PMC5378312

[38] Liu J Zhou G Wang X Liu D. Metabolic reprogramming consequences of sepsis: adaptations and contradictions. Cell Mol Life Sci. (2022) 79:456. doi: 10.1007/s00018-022-04490-0, 35904600 PMC9336160

[39] Preau S Vodovar D Jung B Lancel S Zafrani L Flatres A . Energetic dysfunction in sepsis: a narrative review. Ann Intensive Care. (2021) 11:104. doi: 10.1186/s13613-021-00893-7, 34216304 PMC8254847

[40] Hong G Zheng D Zhang L Ni R Wang G Fan GC . Administration of nicotinamide riboside prevents oxidative stress and organ injury in sepsis. Free Radic Biol Med. (2018) 123:125–37. doi: 10.1016/j.freeradbiomed.2018.05.073, 29803807 PMC6236680

[41] Liang H Ding X Li L Wang T Kan Q Wang L . Association of preadmission metformin use and mortality in patients with sepsis and diabetes mellitus: a systematic review and meta-analysis of cohort studies. Crit Care. (2019) 23:50. doi: 10.1186/s13054-019-2346-4, 30777119 PMC6379943

[42] Li X Zeng Q Yao R Zhang L Kong Y Shen B. Rapamycin mitigates organ damage by autophagy-mediated NLRP3 inflammasome inactivation in sepsis. Histol Histopathol. (2024) 39:1167–77. doi: 10.14670/HH-18-706, 38288570

[43] Hendrickson CM Matthay MA. Endothelial biomarkers in human sepsis: pathogenesis and prognosis for ARDS. Pulm Circ. (2018) 8:1–12. doi: 10.1177/2045894018769876, 29575977 PMC5912282

[44] Vincent JL Ramesh MK Ernest D LaRosa SP Pachl J Aikawa N . A randomized, double-blind, placebo-controlled, phase 2b study to evaluate the safety and efficacy of recombinant human soluble thrombomodulin, ART-123, in patients with sepsis and suspected disseminated intravascular coagulation. Crit Care Med. (2013) 41:2069–79. doi: 10.1097/CCM.0b013e31828e9b03, 23979365

[45] David S Mukherjee A Ghosh CC Yano M Khankin EV Wenger JB . Angiopoietin-2 may contribute to multiple organ dysfunction and death in sepsis. Crit Care Med. (2012) 40:3034–41. doi: 10.1097/CCM.0b013e31825fdc31, 22890252 PMC3705559

[46] Zhuo M Fu S Chi Y Li X Li S Ma X . Angiopoietin-2 as a prognostic biomarker in septic adult patients: a systemic review and meta-analysis. Ann Intensive Care. (2024) 14:169. doi: 10.1186/s13613-024-01393-0, 39522088 PMC11551087

[47] Martelli D McKinley MJ McAllen RM. The cholinergic anti-inflammatory pathway: a critical review. Auton Neurosci. (2014) 182:65–9. doi: 10.1016/j.autneu.2013.12.007 24411268

[48] Morelli A Ertmer C Westphal M Rehberg S Kampmeier T Ligges S . Effect of heart rate control with esmolol on hemodynamic and clinical outcomes in patients with septic shock: a randomized clinical trial. JAMA. (2013) 310:1683–91. doi: 10.1001/jama.2013.278477, 24108526

[49] Rehberg S Frank S Černý V Cihlář R Borgstedt R Biancofiore G . Landiolol for heart rate control in patients with septic shock and persistent tachycardia. A multicenter randomized clinical trial (Landi-SEP). Intensive Care Med. (2024) 50:1622–34. doi: 10.1007/s00134-024-07587-1, 39297945 PMC11447033

[50] Whitehouse T Hossain A Perkins GD Gordon AC Bion J Young D . Landiolol and organ failure in patients with septic shock: the STRESS-L randomized clinical trial. JAMA. (2023) 330:1641–52. doi: 10.1001/jama.2023.20134 37877587 PMC10600724

[51] Komorowski M Celi LA Badawi O Gordon AC Faisal AA. The artificial intelligence clinician learns optimal treatment strategies for sepsis in intensive care. Nat Med. (2018) 24:1716–20. doi: 10.1038/s41591-018-0213-5, 30349085

[52] Gholami B Haddad WM Bailey JM Muir WW. Closed-loop control for fluid resuscitation: recent advances and future challenges. Front Vet Sci. (2021) 8:642440. doi: 10.3389/fvets.2021.642440, 33708814 PMC7940185

[53] Gupta CB Basu D Williams TK Neff LP Johnson MA Patel NT . Improving the precision of shock resuscitation by predicting fluid responsiveness with machine learning and arterial blood pressure waveform data. Sci Rep. (2024) 14:2227. doi: 10.1038/s41598-023-50120-5, 38278825 PMC10817926

[54] Torres-Arrese M García-Casasola G Blancas R. Role of point-of-care ultrasound in septic shock. Med Clin. (2026) 166:107269. doi: 10.1016/j.medcli.2025.107269, 41505938

[55] Leijte GP Rimmelé T Kox M Bruse N Monard C Gossez M . Monocytic HLA-DR expression kinetics in septic shock patients with different pathogens, sites of infection and adverse outcomes. Crit Care. (2020) 24:110. doi: 10.1186/s13054-020-2830-x, 32192532 PMC7082984

[56] Antcliffe DB Harte E Hussain H Jiménez B Browning C Gordon AC. Metabolic septic shock sub-phenotypes, stability over time and association with clinical outcome. Intensive Care Med. (2025) 51:529–41. doi: 10.1007/s00134-025-07859-4, 40163132 PMC12018528

[57] Muralitharan S Nelson W Di S McGillion M Devereaux PJ Barr NG . Machine learning-based early warning systems for clinical deterioration: systematic scoping review. J Med Internet Res. (2021) 23:e25187. doi: 10.2196/25187, 33538696 PMC7892287

[58] Seymour CW Liu VX Iwashyna TJ Brunkhorst FM Rea TD Scherag A . Assessment of clinical criteria for Sepsis: for the third international consensus definitions for Sepsis and septic shock (Sepsis-3). JAMA. (2016) 315:762–74. doi: 10.1001/jama.2016.0288, 26903335 PMC5433435

[59] Johnson AE Pollard TJ Shen L Lehman LW Feng M Ghassemi M . MIMIC-III, a freely accessible critical care database. Sci Data. (2016) 3:160035. doi: 10.1038/sdata.2016.35, 27219127 PMC4878278

[60] Yao YM Zhang H Dong N. Classification of sepsis: the cornerstone for precise treatment. Chin J Burns Wounds. (2024) 40:915–9. doi: 10.3760/cma.j.cn501225-20240529-00203

[61] Moore AR Zheng H Ganesan A Hasin-Brumshtein Y Maddali MV Levitt JE . A consensus immune dysregulation framework for sepsis and critical illnesses. Nat Med. (2025) 31:4084–96. doi: 10.1038/s41591-025-03956-5, 41028543 PMC12705441

[62] Ge Q Song W Ding H Wu H Ren Z. Dynamic changes and prognostic utility of procalcitonin, D-dimer, and lactate dehydrogenase in patients with sepsis and septic shock. Front Med. (2026) 13:1771448. doi: 10.3389/fmed.2026.1771448, 41846879 PMC12989606

[63] Kijpaisalratana N Saoraya J Nhuboonkaew P Vongkulbhisan K Musikatavorn K. Real-time machine learning-assisted sepsis alert enhances the timeliness of antibiotic administration and diagnostic accuracy in emergency department patients with sepsis: a cluster-randomized trial. Intern Emerg Med. (2024) 19:1415–24. doi: 10.1007/s11739-024-03535-5, 38381351

[64] Liu S See KC Ngiam KY Celi LA Sun X Feng M. Reinforcement learning for clinical decision support in critical care: comprehensive review. J Med Internet Res. (2020) 22:e18477. doi: 10.2196/18477, 32706670 PMC7400046

[65] Wake DT Smith DM Kazi S Dunnenberger HM. Pharmacogenomic clinical decision support: a review, how-to guide, and future vision. Clin Pharmacol Ther. (2022) 112:44–57. doi: 10.1002/cpt.2387, 34365648 PMC9291515

[66] Topol EJ. High-performance medicine: the convergence of human and artificial intelligence. Nat Med. (2019) 25:44–56. doi: 10.1038/s41591-018-0300-7, 30617339

[67] Obermeyer Z Powers B Vogeli C Mullainathan S. Dissecting racial bias in an algorithm used to manage the health of populations. Science. (2019) 366:447–53. doi: 10.1126/science.aax2342, 31649194

[68] Kelly CJ Karthikesalingam A Suleyman M Corrado G King D. Key challenges for delivering clinical impact with artificial intelligence. BMC Med. (2019) 17:195. doi: 10.1186/s12916-019-1426-2, 31665002 PMC6821018

[69] Doshi-Velez F Kim B. Towards a rigorous science of interpretable machine learning. [Epubh ahead of preprint] (2017). doi: 10.48550/arXiv.1702.08608.

[70] Woodcock J LaVange LM. Master protocols to study multiple therapies, multiple diseases, or both. N Engl J Med. (2017) 377:62–70. doi: 10.1056/NEJMra1510062, 28679092

[71] Angus DC Berry S Lewis RJ Al-Beidh F Arabi Y van Bentum-Puijk W . The REMAP-CAP (randomized embedded multifactorial adaptive platform for community-acquired pneumonia) study. Rationale and design. Ann Am Thorac Soc. (2020) 17:879–91. doi: 10.1513/AnnalsATS.202003-192SD, 32267771 PMC7328186

[72] Antcliffe DB Burnham KL Al-Beidh F Santhakumaran S Brett SJ Hinds CJ . Transcriptomic signatures in sepsis and a differential response to steroids. From the VANISH randomized trial. Am J Respir Crit Care Med. (2019) 199:980–6. doi: 10.1164/rccm.201807-1419OC, 30365341 PMC6467319

[73] Laterre PF Pickkers P Marx G Wittebole X Meziani F Dugernier T . Safety and tolerability of non-neutralizing adrenomedullin antibody adrecizumab (HAM8101) in septic shock patients: the AdrenOSS-2 phase 2a biomarker-guided trial. Intensive Care Med. (2021) 47:1284–94. doi: 10.1007/s00134-021-06537-5, 34605947 PMC8487806

[74] Geven C Blet A Kox M Hartmann O Scigalla P Zimmermann J . A double-blind, placebo-controlled, randomised, multicentre, proof-of-concept and dose-finding phase II clinical trial to investigate the safety, tolerability and efficacy of adrecizumab in patients with septic shock and elevated adrenomedullin concentration (AdrenOSS-2). BMJ Open. (2019) 9:e024475. doi: 10.1136/bmjopen-2018-024475, 30782906 PMC6377571

[75] Llitjos JF Carrol ED Osuchowski MF Bonneville M Scicluna BP Payen D . Enhancing sepsis biomarker development: key considerations from public and private perspectives. Crit Care. (2024) 28:238. doi: 10.1186/s13054-024-05032-9, 39003476 PMC11246589

[76] Giamarellos-Bourboulis EJ Aschenbrenner AC Bauer M Bock C Calandra T Gat-Viks I . The pathophysiology of sepsis and precision-medicine-based immunotherapy. Nat Immunol. (2024) 25:19–28. doi: 10.1038/s41590-023-01660-5, 38168953

[77] Gordon AC Alipanah-Lechner N Bos LD Dianti J Diaz JV Finfer S . From ICU syndromes to ICU subphenotypes: consensus report and recommendations for developing precision medicine in the ICU. Am J Respir Crit Care Med. (2024) 210:155–66. doi: 10.1164/rccm.202311-2086SO, 38687499 PMC11273306

[78] Khoury MJ Bowen S Dotson WD Drzymalla E Green RF Goldstein R . Health equity in the implementation of genomics and precision medicine: a public health imperative. Genet Med. (2022) 24:1630–9. doi: 10.1016/j.gim.2022.04.009, 35482015 PMC9378460

[79] Green S Prainsack B Sabatello M. The roots of (in)equity in precision medicine: gaps in the discourse. Per Med. (2024) 21:5–9. doi: 10.2217/pme-2023-0097, 38088178 PMC10784620

[80] Ginsburg GS Phillips KA. Precision medicine: from science to value. Health Aff Millwood. (2018) 37:694–701. doi: 10.1377/hlthaff.2017.1624, 29733705 PMC5989714

[81] Kasztura M Richard A Bempong NE Loncar D Flahault A. Cost-effectiveness of precision medicine: a scoping review. Int J Public Health. (2019) 64:1261–71. doi: 10.1007/s00038-019-01298-x, 31650223 PMC6867980

[82] Phillips KA Ann Sakowski J Trosman J Douglas MP Liang SY Neumann P. The economic value of personalized medicine tests: what we know and what we need to know. Genet Med. (2014) 16:251–7. doi: 10.1038/gim.2013.122, 24232413 PMC3949119

[83] Dzau VJ Ginsburg GS. Realizing the full potential of precision medicine in health and health care. JAMA. (2016) 316:1659–60. doi: 10.1001/jama.2016.14117, 27669484

